# Trendy e-cigarettes enter Europe: chemical characterization of JUUL pods and its aerosols

**DOI:** 10.1007/s00204-020-02716-3

**Published:** 2020-03-18

**Authors:** Nadja Mallock, Hai Linh Trieu, Miriam Macziol, Sebastian Malke, Aaron Katz, Peter Laux, Frank Henkler-Stephani, Jürgen Hahn, Christoph Hutzler, Andreas Luch

**Affiliations:** 1 Department of Chemical and Product Safety, German Federal Institute for Risk Assessment (BfR), Berlin, Germany; 2 Department of Biology, Chemistry, Pharmacy, Institute of Pharmacy, Freie Universität Berlin, Berlin, Germany; 3 Official Chemical and Veterinary Surveillance Institute Sigmaringen (CVUA), Sigmaringen, Germany

**Keywords:** JUUL, Electronic cigarette, Nicotine salt, Nicotine delivery, Vapor chemistry, Benzoic acid

## Abstract

The popularity and the high nicotine content of the American pod e-cigarette JUUL have raised many concerns. To comply with European law, the nicotine concentration in the liquids of the European version, which has been recently released on the market, is limited to below 20 mg/mL. This limit can possibly be circumvented by technological adjustments that increase vaporization and consequently, elevate nicotine delivery. In this study, we compare vapor generation and nicotine delivery of the initial European version, a modified European version, and the original American high-nicotine variant using a machine vaping set-up. Additionally, benzoic acid and carbonyl compounds are quantified in the aerosol. Further, concentrations of nicotine, benzoic acid, propylene glycol, and glycerol, along with the density and pH value of JUUL e-liquids have been assessed. Whereas the initial European version did not compensate for the low nicotine content in the liquid, we provide evidence for an increased vaporization by the modified European version. As a consequence, nicotine delivery per puff approximates the American original. Notably, this is not associated with an increased generation of carbonyl compounds. Our data suggest a similar addictiveness of the enhanced European version and the original American product.

**Electronic supplementary material:**

The online version of this article (10.1007/s00204-020-02716-3) contains supplementary material, which is available to authorized users.

## Introduction

Electronic cigarettes have been and are still at the center of controversies among researchers and policy makers. There is growing consensus that the exposure to carcinogens and hence toxicological risks are markedly reduced when compared to combustible tobacco cigarettes (Stephens 2018), where combustion and pyrolysis of organic material lead to the formation of toxicologically relevant substances (Rodgman and Green 2003). Yet, carbonyl compounds, such as acetaldehyde or formaldehyde, have also been found in e-cigarette vapor under dry-puff conditions. When used under normal conditions, much lower levels are detected compared to cigarette smoke though (Farsalinos and Gillman 2018; Goniewicz et al. 2014; Hutzler et al. 2014). Carbonyls can be formed from the liquid components glycerol (vegetable glycerol, VG) or propylene glycol (PG) by thermal decomposition (Gillman et al. 2016; Paine et al. 2007). On the other hand, novel risks may arise from constituents that are untypical for conventional cigarettes (Erythropel et al. 2019b; Kaur et al. 2018). Despite the confirmed reduction of the long known and established toxicants, possible health disturbances of e-cigarette consumption, such as the impact of nicotine on brain development (Dwyer et al. 2008; Smith et al. 2015) and the cardiovascular system (Buchanan et al. 2019), need to be further investigated and assessed. Conversely, also assumed benefits on the population level need to be clarified. For example, it is criticized that smokers who switch to e-cigarettes do not quit nicotine consumption but use e-cigarettes for a longer term. A gateway effect is debated, but more data are required to clarify this assumption (Conner et al. 2018; Etter 2018; Kandel and Kandel 2015; Liu et al. 2020; Watkins et al. 2018).

The product spectrum of e-cigarettes is rapidly expanding. Currently, there are two major developments in the field: First, “open systems” that allow the consumer to adjust vaporization power settings and the nicotine concentration in the liquid individually, and second, “pod systems” that already contain the coil, the wick and a small liquid reservoir. These latter devices are comparatively simple and easy to use. Pod systems usually contain highly concentrated nicotine salt formulations and have recently been demonstrated to deliver nicotine nearly equally efficient as combustible cigarettes (Bowen and Xing 2014; O'Connell et al. 2019). The high nicotine content of up to 5% (approximately 58 mg/mL), the resulting high pH value of the vapor and the high proportion of free-base nicotine would normally lead to airway irritation (Duell et al. 2018). Weak organic acids, such as benzoic or salicylic acid, are being supplemented to adjust the pH value and the amount of free-base nicotine to a level that is more tolerable for the consumer (Bowen and Xing 2014; Duell et al. 2018).

E-liquids of the brand JUUL contain benzoic acid and high nicotine concentrations of up to 5%. The brand had reached a market share of about 40% in the US by the end of 2017 (Huang et al. 2019) with high popularity among adolescents (Hammond et al. 2019; Krishnan-Sarin et al. 2019). This is likely due to viral marketing and the spreading of the product via social media and YouTube (Allem et al. 2018; Brett et al. 2019; Chu et al. 2018; Czaplicki et al. 2019; Kavuluru et al. 2019; Ramamurthi et al. 2019). The product design is flat and the vapor generation is low, which allows unsuspicious “stealth” vaping that even went viral as an internet challenge (Ramamurthi et al. 2019). Evidence for nicotine dependence in adolescent pod users has been shown in a pilot study (Boykan et al. 2019). The recent e-cigarette innovations have led to a “public health epidemic” in the US, as stated by the Surgeon General (U.S. Surgeon General 2018).

In 2018, JUUL was also introduced in Germany and was required to comply with the European Tobacco Product Directive 2014/40/EU (TPD), in respect to the upper limit of 20 mg/mL for nicotine (European Parliament 2014). Consequently, a lower nicotine delivery could be expected for the European version, but no data are yet available. Some research groups have already characterized the US-American variant (Duell et al. 2018; Erythropel et al. 2019a; Pankow et al. 2017; Reilly et al. 2019; Talih et al. 2019). The nicotine content in the aerosol was comparable to combustible cigarettes, but formation of carbonyl compounds was low as expected for low power vaporizers (Reilly et al. 2019; Talih et al. 2019). We have hypothesized that the manufacturer might increase the vapor generation of the European version to compensate for the low nicotine content in the liquid. In fact, an improved version of European JUUL, referred to as “Turbo” by JUUL employees (Mahase 2019), has been recently introduced in Germany and prompted concerns of an increased addictiveness. In this study, we aim to set a reference point for nicotine and toxicant levels in the aerosol of the initial and the modified version of JUUL. Technical aspects for vaping machine experiments are also briefly discussed. Therefore this study provides a scientific ground for the monitoring of current and further directions of product development.

## Methods

### E-cigarettes and pods

The European devices and differently flavored pods were purchased in local stores in Berlin and Sigmaringen, Germany, and online. The US-American variant was purchased in Tempe, Arizona.

### Chemicals and standard substances

Used solvents or chemicals were of analytical or higher purity grade. 2-Propanol containing 0.3 g/L *n*-heptadecane, 2 g/L ethanol as internal standards and (*S*)-nicotine salicylate were purchased from LGC Standards (Teddington, UK), acetonitrile, sodium chloride, and orthophosphoric acid (85%) from Merck KGaA (Darmstadt, Germany). 2,4-Dinitrophenylhydrazine (moistened with 33% water) was bought from PanReac AppliChem (Darmstadt, Germany). Tris(hydroxymethyl)aminomethane, sulfuric acid (99.999%), (*S*)-nicotine, benzoic acid, benzoic acid-d_5_, and the 2,4-dinitrophenylhydrazone (DNPH) derivatives of acetaldehyde, acetone, acrolein, and formaldehyde were purchased from Sigma-Aldrich (Taufkirchen, Germany). Dimethyl sulfoxide was purchased from Honeywell Riedel-de-Haën (Seelze, Germany). Ultrapure water was prepared with a Milli-Q Integral Water Purification System (Merck KGaA, Darmstadt, Germany).

### Aerosol generation

Aerosols were generated in two different laboratories, hereafter referred to as “lab A” (BfR, Berlin, Germany) and “lab B” (CVUA Sigmaringen, Sigmaringen, Germany). Both laboratories used a standard linear smoking machine that was designed for e-cigarettes (LM4E with PM1 piston pump, Borgwaldt, Hamburg, Germany). Experiments were performed according to CORESTA Reference Method 81 (CORESTA 2015) for the puffing regimen: 55 mL puff volume, 3 s puff duration, 30 s puff frequency, and rectangular puffing profile. E-cigarettes were placed in an angle of − 15° from a horizontal position. Except for carbonyl analysis, sessions of 20 puffs were taken with a clearing puff without e-cigarette at the end of each session. Between sessions, the liquid was allowed to cool down for approximately 10 min. The batteries were recharged after 8 and 6 sessions for the initial and the modified pods, respectively. Lab A compared two different custom adapters for a tight placement of the angular shaped e-cigarette mouth-piece on the filter holders as displayed in Fig. 5 in the Supplementary Material. One adapter was self-made with a heat shrinkable tubing (cross-linked polyolefin, HStronic GmbH, Schwäbisch Hall, Germany) that was prepared once and reused in combination with tape sealing (Parafilm, Bemis Company, Neenah, WI, USA). The second adapter was purchased from Borgwaldt (Hamburg, Germany) and used without additional tape sealing. Lab B used only the mouth-piece from Borgwaldt.

### Determination of liquid consumption and total particulate matter (TPM), water and nicotine in the aerosol

TPM was collected on Ø 44 cm glass fiber filters (Borgwaldt, Hamburg, Germany). The filters in the filter holders and the e-cigarettes with pods were weighed before and after each session on analytical scales (LE225-0CE in lab A and CP225D-0CE in lab B, both Sartorius, Göttingen, Germany). TPM was calculated with the weight gain of the filters according to ISO 4387 (2019), consumption of the e-liquid with the weight loss of the liquid. Filters were extracted with 20 mL isopropanol containing internal standards (0.3 mg/mL *n*-heptadecane, 2 mg/mL ethanol) on a horizontal shaker (lab A: 3005, GFL, Burgwedel, Germany; lab B: SM-30 Control, Edmund Bühler, Hechingen, Germany) for 30 min at 80–100 rpm. The extracts were used for the determination of nicotine and water. Nicotine was quantified by gas chromatography with flame ionization detection (GC/FID). Lab A used a 6890 series from Agilent Technologies/Hewlett Packard (Agilent Technologies, Waldbronn, Germany) with a constant flow of 1.3 mL/min hydrogen (purity 99.999%, Linde, Pullach, Germany) on an HP-5 ms capillary column (30 m length, 250 µm inner diameter, 0.25 µm film thickness, 3 m pre-column, Agilent Technologies, Waldbronn, Germany). The temperature program started with 5 min at 100 °C, followed by a 30 °C/min ramp to 325 °C with 3.50 min hold. 1 µL filter extract was injected into a split/splitless injector at 250 °C and split ratio of 1:5 was used. FID was operated at 290 °C with a hydrogen flow of 30 mL/min, air flow of 300 mL/min, and a nitrogen (purity 99.999%, Linde, Pullach, Germany) make up flow of 20 mL/min. Lab B analyzed nicotine with flame ionization detection at 300 °C (7890A, Agilent Technologies, Waldbronn, Germany; 30 mL/min H_2_ flow, 99.999%; 400 mL/min air flow; 15 mL/min make up flow, N_2_, 99.999%; Air Liquide, Paris, France) and water with thermal conductivity detection at 250 °C (Agilent Technologies, Waldbronn, Germany; 15.5 mL/min reference flow, 5 mL/min combined flow) in one run after separation with a 7890A series gas chromatograph (Agilent Technologies, Waldbronn, Germany). 1 µL extract was injected into a split/splitless injector at 250 °C in splitless mode. Separation for nicotine analysis was performed by using an Rtx-VMS column (30 m × 0.530 mm, 3 µm film thickness, Restek GmbH, Bad Homburg, Germany), and for water an HP Plot Q column (30 m × 0.530 mm, 45 µm film thickness, Agilent Technologies, Waldbronn, Germany).The oven program started at 75 °C for 0.5 min, heated with a rate of 50 °C/min to 165 °C and held for 3 min, heated with 50 °C/min to 225 °C, held for 5 min, before it cooled down at 50 °C/min to 75 °C, followed by a 1 min hold. Flow rate of helium carrier gas (99.999%, Air Liquide, Paris, France) was 4.240 mL/min.

### Determination of carbonyl compounds

Carbonyls were analyzed as described previously (Mallock et al. 2018) with liquid chromatography and UV detection on an RP-Amid column (Ascentis, 150 × 2 mm, 3 µm, Supelco, Bellefonte, PA, USA). Fractions of 40 puffs each were drawn through impingers that contained 35 mL of 2,4-dinitrophenylhydrazine (3.4 mg/mL in 45% acetonitrile with 0.35% orthophosphoric acid) each. After two clearing puffs, the content of both impingers was combined and incubated at room temperature for 30 min before reaction was stopped by addition of 2 mL tris(hydroxymethyl)aminomethane (tris) solution (16 mg/mL in 80% acetonitrile) to 8 mL of the sample. Calibration standards for carbonyl-DNPH-derivatives were diluted with the same DNPH/tris solution mixture to mimic effects of excess DNPH on the UV spectra.

### Determination of benzoic acid in liquids and aerosol

Benzoic acid was quantified using headspace-solid phase microextraction-gas chromatography/mass spectrometry (HS-SPME-GC/MS). 60 mg of sample liquid or self-prepared standard liquid (20 mg/mL nicotine in PG/VG 50:50 (w/w)) was weighed into 20 mL headspace vials and dissolved in 5 mL saturated sodium chloride solution containing 0.5 M sulfuric acid. 50 µL of isotope-labeled internal standard solution (8 mg/mL benzoic acid-d_5_) and/or calibration standard solution (4, 10, 15, 20 and 30 mg/mL benzoic acid) in DMSO were added and mixed. For analysis of benzoic acid in the aerosol, vaped filters were transferred into 20 mL headspace vials. For calibration, blank filters were used in combination with 40 mg or 80 mg standard liquid. Standard solutions were directly pipetted on the filter, followed by mixing with 5 mL saturated salt solution containing 0.5 M sulfuric acid. SPME was automated on an MPS2-XL autosampler (Gerstel, Mühlheim, Germany) with an incubation temperature of 80 °C and 1 min incubation time prior to 50 min headspace extraction by a polydimethylsiloxane/divinylbenzene fiber (Supelco, Bellafonte, PA, USA) with 250 rpm shaking only for the filter samples. The fiber was injected into a cooled injection system (CIS) 4 (Gerstel, Mühlheim, Germany) and desorbed for 5 min at 250 °C and a 1:50 split ratio. The GC 6890A (Agilent Technologies, Waldbronn, Germany) was equipped with a 30 m HP-FFAP capillary column with 250 µm inner diameter and 0.25 µm film thickness (Agilent Technologies, Waldbronn, Germany). After 5.5 min at 60 °C, the GC oven ramped with 15 °C/min to 240 °C and held for 15 min. The helium (purity 99.999%, Linde, Pullach, Germany) carrier gas flow was constant at 1 mL/min. The mass selective detector MSD 5975C (Agilent Technologies, Waldbronn, Germany) was equipped with an electron impact ion source (Agilent Technologies, Waldbronn, Germany) and operated with an ionization energy of 70 eV using a combined selected ion monitoring (SIM) and scan mode with a mass range from 29 to 300 m/z. Benzoic acid was quantified with the ion masses of 77 m/z and qualified with 105 m/z and 122 m/z. The internal standard benzoic acid-d_5_ was quantified with 82 m/z and qualified with 110 m/z and 127 m/z. Dwell time was 15 ms for each ion. Optimization of extraction parameters is summarized in the Supplementary Material.

### Determination of density, pH value and nicotine content of the e-liquid

Liquids from the same batch were pooled for direct determination of density with an oscillating U-tube (DMA 500, Anton Paar, Graz, Austria). For quantification of the nicotine content in liquids, 300 mg liquid was diluted in 10 mL isopropanol with internal standards (0.3 mg/mL *n*-heptadecane, 2 mg/mL ethanol) and analyzed with the above mentioned GC/FID method in lab B. The pH value of a 1:20 dilution of liquids in ultrapure water was directly measured with a pH meter (765 Calimatic; Knick, Berlin, Germany).

### Determination of propylene glycol and glycerol content of the e-liquid

E-liquids were analyzed by diluting a sample solution of approx. 5 mg/mL (precisely weighed) with methanol. The resulting solution was diluted by 1:1 with the internal standard solution containing 5 mg/mL 1,4-butanediol in methanol. 1 µL aliquot of this sample solution was injected into the split/splitless injector and analyzed by means of GC/FID. GC/FID analysis was performed on an Agilent 7890A gas chromatograph equipped with an FID detector and an autosampler (Agilent Technologies, Waldbronn, Germany). Separation was achieved on an HP-FFAP (25 m × 0.32 mm i.d. × 0.52 μm film) capillary column (Agilent Technologies, Waldbronn, Germany). GC/FID conditions were as follows: split mode, split ratio: 1:40; injector temperature: 230 °C; nitrogen (99.999%; Air Liquide, Paris, France) as carrier gas at a constant pressure of 0.7 bar. FID was operated at 250 °C (30 mL/min H_2_ flow, 99.999%; 400 mL/min air flow; 30 mL/min make up flow, N_2_, 99.999%; Air Liquide, Paris, France). The oven program started at 70 °C, held for 4 min. The temperature was raised by 10 °C/min up to 220 °C and held for 7 min, followed by a ramp of 30 °C/min to 70 °C. Total run time was 31 min.

### Characterization of the pod construction

Resistance was measured between the connectors at the bottom of the pods with a 2010 DMM ohmmeter (PeakTech, Ahrensburg, Germany). For FT-IR analysis, the wick was removed from the pod, washed twice with ethanol, dried at 80 °C (FED 240, Binder, Tuttlingen, Germany), and analyzed with attenuated total reflectance-Fourier-transform infrared (ATR-FTIR) spectroscopy using a Nicolet 6700 spectrometer (Thermo Electron Corporation, Madison, WI).

## Results

### Description of the product

**Fig. 1:**
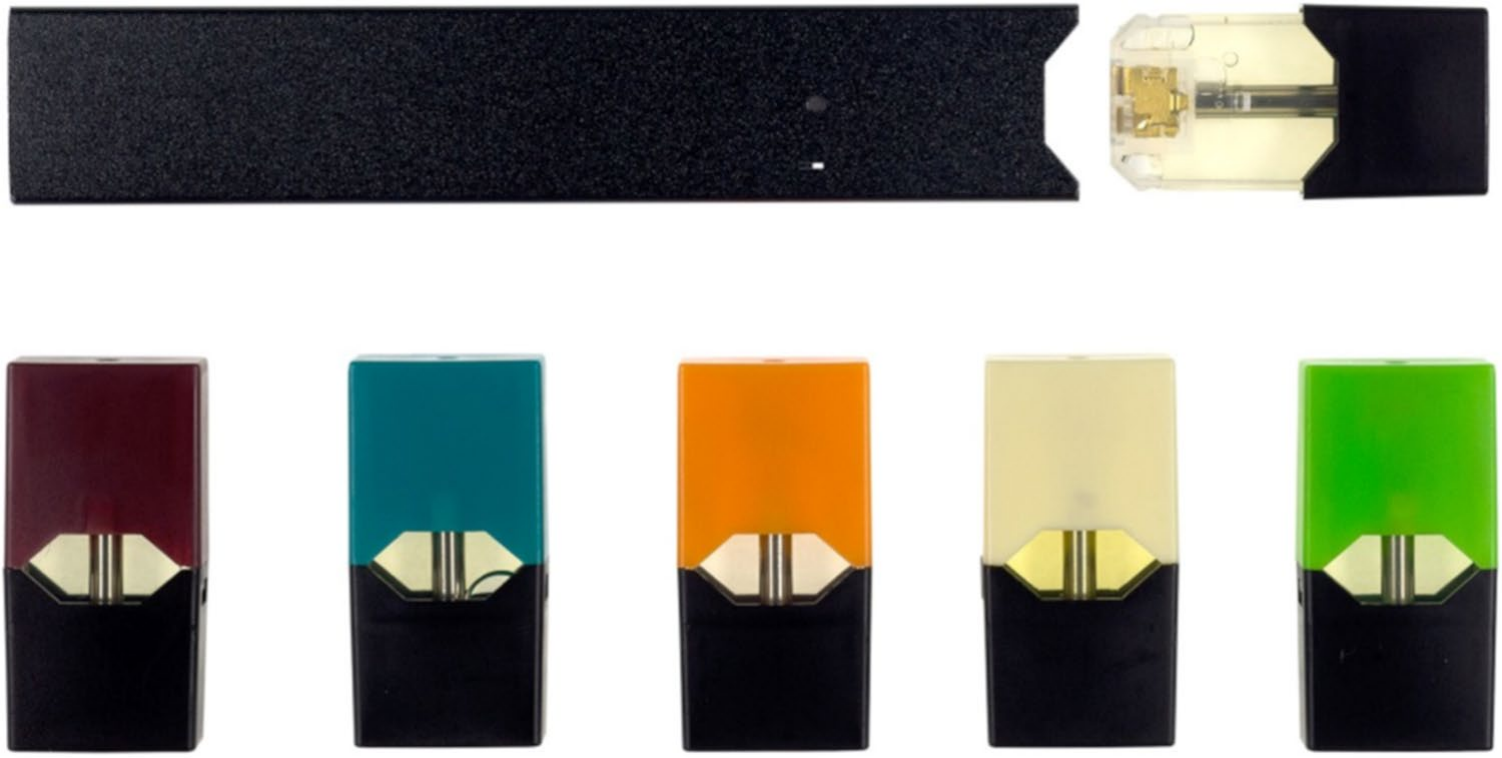
Photography of a JUUL e-cigarette with a battery and differently flavored pods

The JUUL device consists of a flat and elongated battery with contacts to connect to the particular pods as shown in Fig. 1. The prefilled and disposable pods are composed of an e-liquid tank, including coil and wick, and a rectangular mouthpiece. The pods are marketed in four-packs and are declared to contain approximately 0.7 mL liquid with formerly 20 mg/mL (referred to as “initial” in this publication) and now 18 mg/mL and 9 mg/mL nicotine (referred to as “modified”). The modified JUUL version has been launched in Germany in summer 2019, and could still be included in our study. Vegetable glycerol (VG), propylene glycol (PG), nicotine, benzoic acid and “aromas” are listed as ingredients. The packets contain health warnings and hazard pictograms and refer to the product as “alternative for adult smokers”.

### Chemical characterization of JUUL pods and aerosol

Different analytical assessments have been performed in the liquid and the aerosol of JUUL pods with the aromas “Rich Tobacco” (initial and modified pods in comparison), “Royal Creme”, “Mint”, “Mango”, and “Apple” (initial pods). The results are shown in Table 1. Although declared as 20 mg/mL, nicotine content was found to be below 18 mg/mL in the initial pods. Thus, the modified European pods contained the same amount of nicotine as the initial ones. Density, composition of the liquid basis, pH values, and amount of benzoic acid did not vary significantly between different aromas of the initial pods. Modified European JUUL generated more TPM (as marker for vapor generation) as the initial European version. The molar ratio of nicotine to benzoic acid decreased in both liquid and vapor of the improved European version, implying that more benzoic acid is now being applied. The pattern of aldehyde formation changed with the alteration of the pod design: The generation of acetone increased whereas the generation of acetaldehyde and formaldehyde decreased. For formaldehyde, the high standard deviation is likely due to inter- and intra-device variabilities of carbonyl generation. As discussed in the Supplementary Material, the concentrations of all other analytes were close to its analytical thresholds, what could have been an additional factor for high deviations. Furthermore, the amount of water in the vapor has been assessed for 10 initial Rich Tobacco JUUL pods. The mean and standard deviation of the first 160 puffs were 0.25 ± 0.08 mg water/puff.

**Table 1:** Chemical characterization of JUUL liquids and aerosol

Flavor	Rich tobacco	Rich tobacco	Rich tobacco	Royal Creme	Apple	Mango	Mint
Pod design	Initial	Modified	Modified	Initial	Initial	Initial	Initial
Declared nicotine concentration (mg/mL)	20	18	9	20	20	20	20
Characterization of liquids							
Measured nicotine concentration (mg/mL)	17.20 ± 0.13 (3 pods)	17.69 ± 0.09 (3 pods)	9.03 ± 0.14 (3 pods)	17.41 ± 0.05 (3 pods)	17.40 ± 0.28 (3 pods)	17.78 ± 0.14 (3 pods)	17.26 ± 0.21 (3 pods)
Density (g/cm^3^)	1.16	1.16	1.16	1.18	1.18	1.18	1.18
Liquid basis (g/100 g)	PG: 26.0 ± 1.6 VG: 56.8 ± 4.0 (5 pods)	PG: 24.4 ± 2.1 VG: 55.8 ± 4.9 (3 pods)	PG: 27.6 ± 0.1 VG: 61.2 ± 0.8 (3 pods)	PG: 23.8 ± 1.5 VG: 64.7 ± 3.5 (3 pods)	PG: 24.0 ± 1.6 VG: 62.5 ± 4.4 (3 pods)	PG: 24.9 ± 0.2 VG: 65.8 ± 0.7 (3 pod)	PG: 23.6 ± 0.4 VG: 65.6 ± 1.2 (4 pods)
pH value (of 1:20 dilution in ultrapure water)	5.51 (1 pod)	5.42 (1 pod)	5.40 (1 pod)	5.42 (1 pod)	5.74 (1 pod)	5.56 (1 pod)	5.52 (1 pod)
Benzoic acid (mg/mL)	9.64 ± 0.05 (3 pods)	12.67 ± 0.38 (3 pod)	7.02 ± 0.21 (3 pods)	9.24 ± 0.04 (3 pods)	8.82 ± 0.59 (3 pods)	9.24 ± 0.09 (3 pods)	9.17 ± 0.02 (3 pods)
Molar ratio (Nicotine:Benzoic acid)	1:0.7	1:1.0	1:1.0	1:0.7	1:0.7	1:0.7	1:0.7
Characterization of aerosol							
TPM (mg per puff) Mean of the first 160 puffs	1.6 ± 0.4 (20 pods)	3.7 ± 0.7 (6 pods)	3.7 ± 0.7 (6 pods)	1.8 ± 0.5 (2 pods)	1.8 ± 0.3 (2 pods)	1.8 ± 0.3 (2 pods)	1.9 ± 0.3 (2 pods)
Nicotine (µg per puff) Mean of the first 160 puffs	23 ± 5 (20 pods)	61 ± 12 (6 pods)	30 ± 6 (6 pods)	23 ± 7 (2 pods)	23 ± 4 (2 pods)	23 ± 4 (2 pods)	24 ± 4 (2 pods)
Benzoic acid (µg per puff) Mean of the first 160 puffs	21 ± 3 (2 pods)	41 ± 6 (2 pods)	22 ± 3 (2 pods)				
Acetaldehyde (ng per puff) Mean of the first 160 puffs	76 ± 116 (4 pods)	12 ± 13 (4 pods)					
Acetone (ng per puff) Mean of the first 160 puffs	3 ± 2 (4 pods)	36 ± 10 (4 pods)					
Acrolein (ng per puff) Mean of the first 160 puffs	13 ± 7 (4 pods)	7 ± 2 (4 pods)					
Formaldehyde (ng per puff) Mean of the first 160 puffs	112 ± 117 (4 pods)	11 ± 6 (4 pods)					

For all aerosol measurements, the commercially available mouth piece was used. Contents of benzoic acid in liquids and vapor, and pH values of liquids were determined in lab A. Density, liquid basis composition, nicotine content of liquids, and carbonyl emissions were analyzed in lab B. TPM and nicotine in the emissions were determined in both labs. Values are presented as mean values and corresponding absolute standard deviations

### Comparison of European JUUL pods with the US-American version

**Fig. 2:**
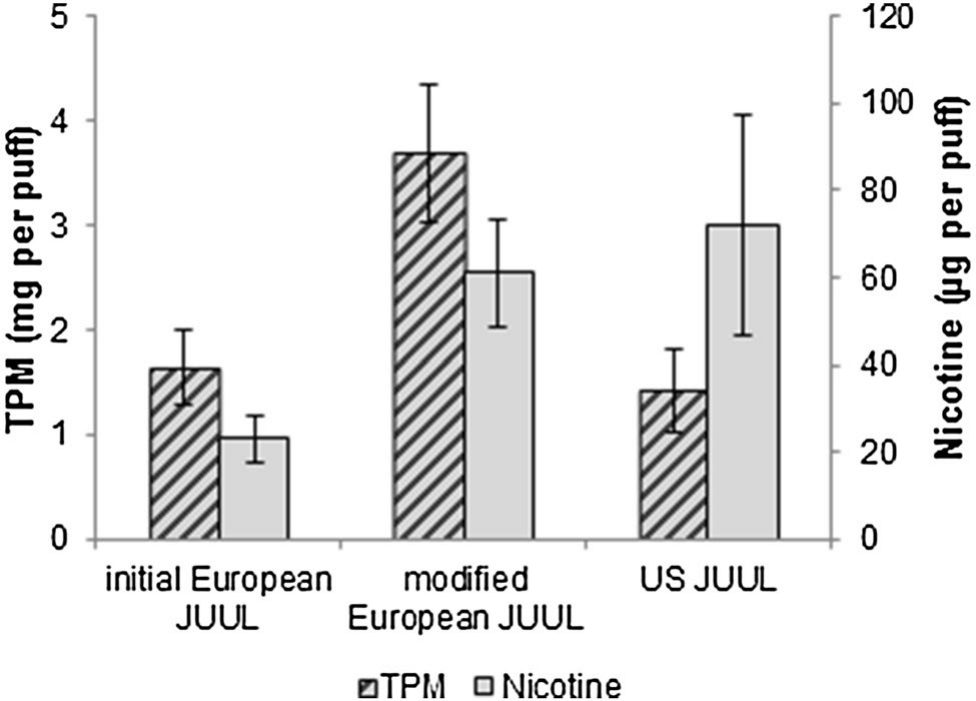
Total particulate matter (TPM) in mg per puff and nicotine levels in µg per puff released during the first 160 puffs of 20 mg/mL initial European Rich Tobacco JUUL (20 pods), 18 mg/mL modified European Rich Tobacco JUUL (6 pods) and 58 mg/mL US-American Virginia Tobacco JUUL (5 pods)

**Fig. 3:**
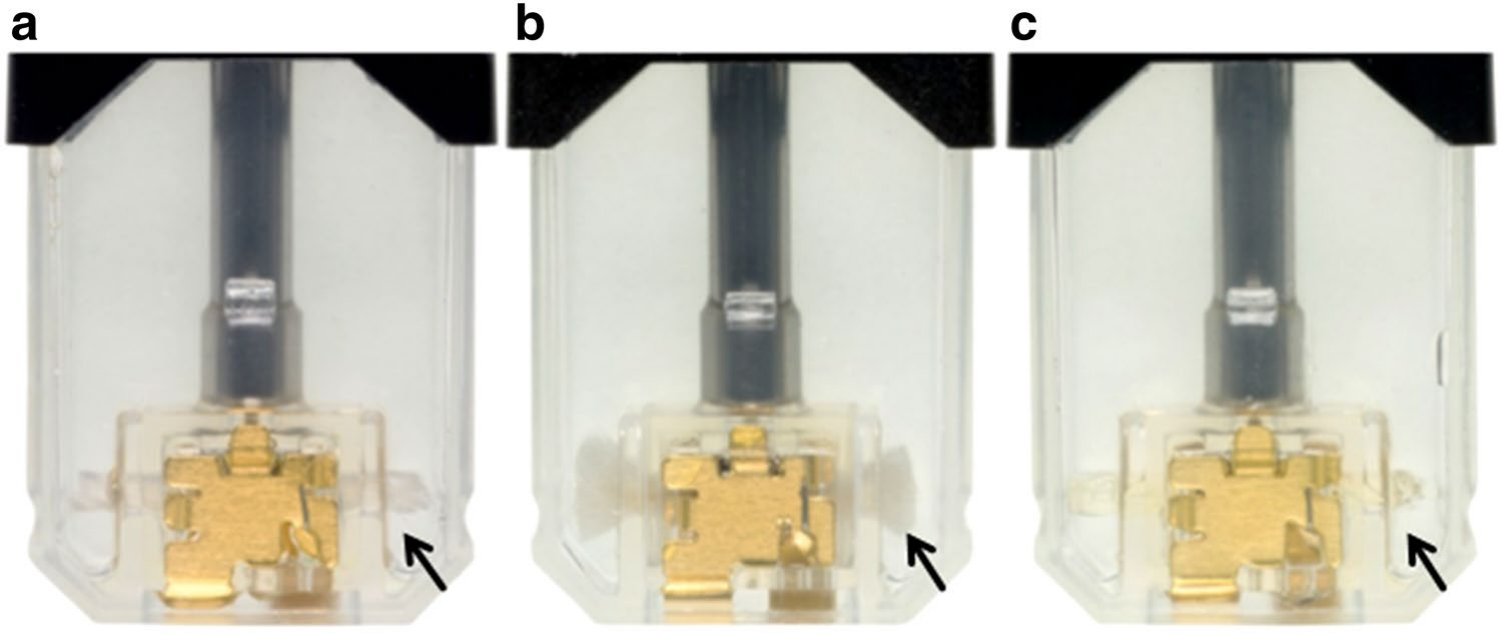
Photographs of emptied JUUL pods: The initial (**a**) and the American (**c**) variant contain a different wick material than the one used in the modified JUUL version (**b**)

As visualized in Fig. 2, the US-American JUUL device released 1.4 ± 0.4 mg TPM per puff, resulting in a similar vapor generation compared to the initial European JUUL. The nicotine delivery was with 72 ± 25 µg per puff approximately threefold higher. This correlates with a threefold higher nicotine content in the liquid. The vapor generation of the European modified JUUL pods was more than doubled compared to both the European initial pods and the US-American version. Accordingly, the nicotine delivery of modified European JUUL approximated to the high-nicotine US-American variant. The resistance between the connections of the pods, reflecting the resistance of the coil, has been measured and ranged between 1.6 and 1.7 Ω for all three variants. As shown in Fig. 3, the wick material has been replaced in the modified JUUL variant. The change in the material has been confirmed with ATR-FTIR. Spectra are displayed in the Supplementary Material.

### Intra-device variability of nicotine delivery

According to European tobacco legislation, E-cigarettes need to deliver nicotine at consistent levels. We have therefore tested whether JUUL complies with this requirement. As illustrated in Figs. 6 and 7 in the Supplementary Material, the aerosol generation of JUUL e-cigarettes varied significantly over all fractions. The continuity of nicotine delivery was assessed in light of the intra-device variability. For each pod analyzed, the mean nicotine delivery for the first 8 fractions was calculated. The difference of each single value to the mean was calculated in percent. The highest difference to the mean was set as maximum deviation for the corresponding pod type in our experiments. This reflects the intra-device variability. Only the first 8 fractions of 20 puffs each were regarded as intended use. Out of 10 initial JUUL pods for each laboratory, the maximum deviation was + 31% and + 79% as determined by lab A and lab B, respectively. Out of the 20 pods, 17 had a maximum deviation above an exemplary threshold of 15% that might be used to define a consistent nicotine delivery. The maximum intra-device deviation out of 6 modified pods was − 50% and − 45% for 18 mg/mL and 9 mg/mL nicotine pods, respectively. For all modified pods analyzed, the maximum deviation was found for the first fraction. All 12 pods had a maximum deviation above 15%. When this fraction was left out from the calculation, the maximum deviation was still characterized as − 45% and − 39% for 18 mg/mL and 9 mg/mL nicotine pods, respectively, with 6 out of 12 pods above 15%.

## Discussion

Nicotine-salt pod e-cigarettes, especially the market leader JUUL, have started a controversy that first emerged in the US. The combination of factors like product design, viral marketing, and the high nicotine contents in liquids and corresponding aerosols have triggered a great popularity especially among young people, thus raising concerns by US-American authorities (Koh and Douglas 2019; U.S. Surgeon General). In December 2018, JUUL e-cigarettes became available in Europe, where the nicotine contents in the liquids had to be lowered to 20 mg/mL in order to comply with European regulation (Art. 20 TPD, (European Parliament 2014)). Little is known about the product variants that were placed on the European market.

Our data demonstrate that the initial product on the European market generated a similar amount of vapor when compared to the American version and subsequently only achieved relatively low levels of nicotine delivered into the aerosol. Amidst our investigation, a new product design, referred to as “Turbo”, was launched (Mahase 2019). We could show that the degree of vaporization in the newly designed product increased more than twice and therefore can be considered sufficient to compensate for the lower nicotine contents in the liquid. This observation confirmed our initial expectation that nicotine delivery will be increased with technical adaptions. Modified JUUL was shown to deliver approximately the same amounts of nicotine as the American version in our machine vaping set-up and thus could potentially lead to blood nicotine levels that are comparable to tobacco cigarettes as well.

The increased vaporization of modified JUUL is linked to the use of another wick material. It is not related to a higher power delivery as parameters like the resistance of the coil and the battery voltage did not change. The properties of the wick can also have a substantial influence on vaporization via the liquid supply rate. A wick made of an expansible material can resupply the coil faster and more stably with unvaporized e-liquid. It is visible by the naked eye that the constitution of the wick has changed. The initial version of the product showed very high intra- and inter-device deviations, especially when more than 200 puffs were drawn. Vapor generation by modified JUUL is more stable; however the deviations are still high and do not attest a good consistency of nicotine delivery. Since both versions contain the same amount of liquid, only half the number of puffs can be drawn from the modified version (see Supplementary Material). Depending on changes in consumption behavior, this could have an influence on cost.

We also would expect an increased addictiveness of the modified JUUL version due to the higher nicotine delivery. Non-smokers who start vaping e-cigarettes with such a high nicotine delivery per puff are at higher risk to become dependent. If these novel design features were combined with higher power settings, nicotine delivery might increase further. In the case of pod systems, our data possibly support the notion that setting a limit for nicotine delivery into the aerosol (per puff) might be more purposeful than liquid nicotine content limits only. To this end, the nicotine delivery limits should be similar or even lower when compared to tobacco cigarettes. Pod systems are very simple; they do not require any prior knowledge as in the case of conventional e-cigarettes and can be bought and used directly. Setting a general limit of aerosol nicotine levels in pod systems would at least protect initiating adolescents who are getting exposed to these products the first time.

E-cigarettes and e-liquids are complex products that undergo steady product development. Therefore, it can be anticipated that further product innovations will occur and current knowledge becomes quickly outdated. This is of special importance for regulators and surveillance authorities who not only need to keep up with future product development but who are also in charge of monitoring the already existing products. The Sisyphean challenge of tobacco control to keep pace with the development on the market is complicated by practical problems, for example the connectivity of e-cigarette mouth pieces of new shapes to the filter holders of vaping machines used for analytical testing. In the Supplementary Material we demonstrate that suitable adapters can be self-made and lead to comparable results as commercially available options. The adjusted adapters made out of a heat-shrinkable material are cheap and uncomplicated in production and could be considered whenever connection of e-cigarettes to the vaping machine is troublesome and no commercial option is available.

Pod systems are usually operated with low electrical power and therefore provide some advantages from a toxicological perspective. We found levels of carbonyl compounds in the respective aerosols lying in a similar range as reported for the American and non-American JUUL devices (Hiraki et al. 2019; Reilly et al. 2019; Talih et al. 2019), but magnitudes lower than those found in tobacco cigarettes (Counts et al. 2005). Also in relation to other e-cigarettes, especially with higher power settings, the carbonyl emissions by JUUL are still comparatively low (El-Hellani et al. 2018; Talih et al. 2017, 2019).

Tobacco smokers who switch completely to e-cigarettes significantly reduce their levels of exposure to known cigarette toxicants, as shown recently in a 5-days trial by the manufacturer (Jay et al. 2019). A closed system device with a low power setting has the advantage that toxicant generation is comparatively low and no easy manipulation by the consumer is feasible as with open systems. Composition of e-liquids can be regulated and monitored better, although this might be undermined by third-party suppliers of pods and refill solutions.

High nicotine delivery might pose an increased risk for adolescents to initiate nicotine use. On the other side, this feature can be beneficial for smokers who intend to reduce harm or attempt cessation. Satisfying nicotine delivery might suppress urges to smoke and prevent dual use or a relapse to tobacco cigarettes. This has not been achieved by older generations of e-cigarettes (Fearon et al. 2018). But it is yet unclear how these high nicotine levels affect complete cessation, considering that at some point e-cigarette use should be ceased as well. Possible harm reduction is also counteracted by dual use of tobacco and electronic cigarettes that might even increase toxicological health risks for vapers (Osei et al. 2019).

While tobacco smokers, who switch completely to e-cigarettes, can reduce their exposure to known tobacco cigarette toxicants and putatively reduce health risks, non-smokers that start with e-cigarettes jeopardize their health and are prone to develop an addictive disorder. Therefore, initiation of e-cigarette consumption is strongly discouraged for non-smokers irrespective of their age.

## Electronic supplementary material

Below is the link to the electronic supplementary material.

Supplementary file1 (DOCX 4313 kb)
