# Supplementary material for: Trendy e-cigarettes enter Europe: chemical characterization of JUUL pods and its aerosols
Source: Arch Toxicol. 2020 Mar 18;94(6):1985–94. doi: 10.1007/s00204-020-02716-3 (PMC7303078; doi:10.1007/s00204-020-02716-3)
Supplement: Supplementary file 1 — Supplementary file1 (DOCX 4313 kb) [file 204_2020_2716_MOESM1_ESM.docx]

Supplementary Material

**HS-SPME method development for determination of benzoic acid in liquids and vapor**

Selection of the separation columns and extraction fibers was based on the literature (Dong et al. 2006; Dong and Wang 2006). For any reliable quantification by HS-SPME-GC/MS, extraction needs to be performed under equilibrium conditions. Therefore, the incubation and extraction parameters were optimized. Different times and temperatures were tested in duplicate and the areas under the curves for benzoic acid and internal standards were compared. Some key optimization experiments are summarized in Figure 4.


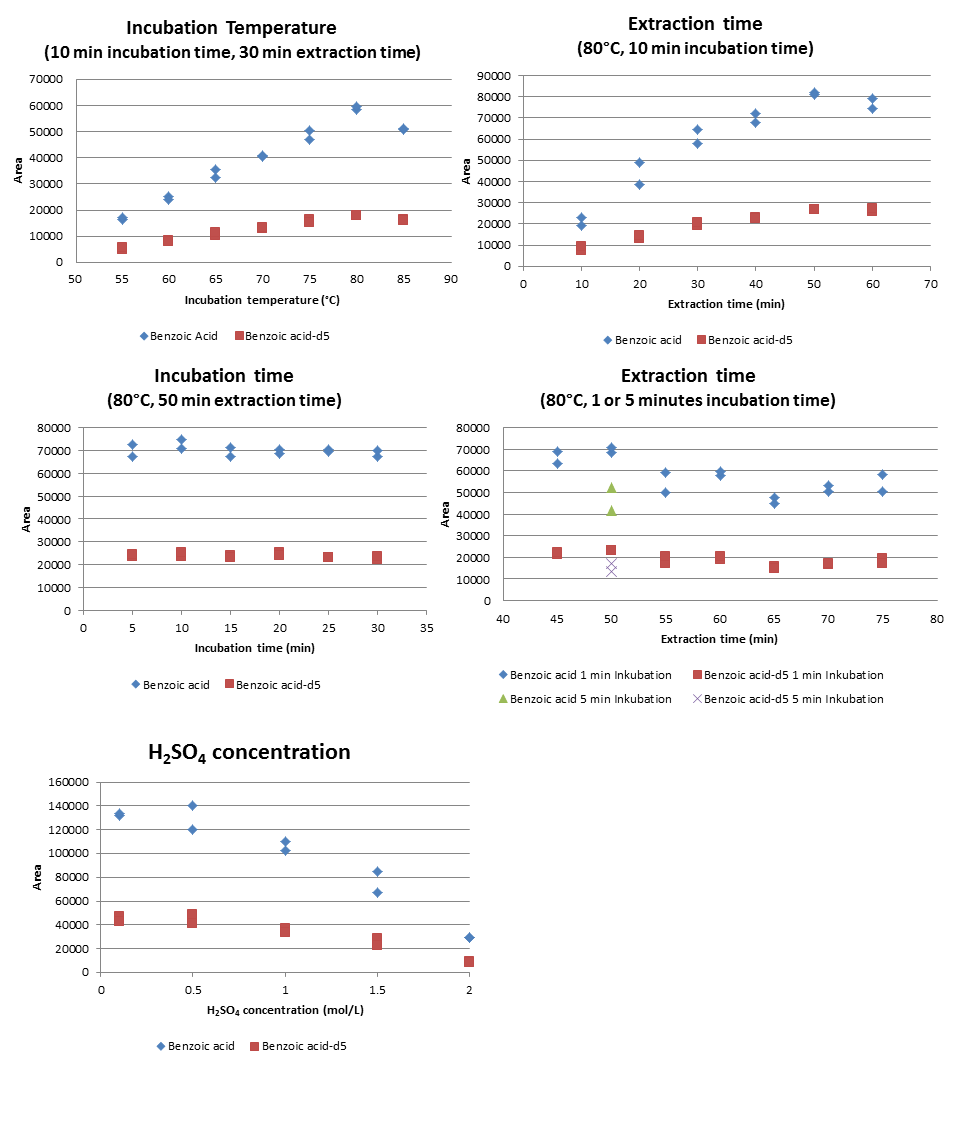


Figure 4. Optimization of Headspace Solid Phase Microextraction (HS-SPME) parameters

**Aerosol generation - Comparison of two mouth pieces by two laboratories**

The rectangular shape of JUUL made it difficult to directly connect the device to the smoking machine using rubber tubes. Our initial solution was the self-fabrication of a fitting mouth piece using a heat-shrinkable tubing (Figure 5b). When the manufacturer of the vaping machine offered a commercially available mouth piece (Figure 5a), we compared the vapor generation using both variants. In lab A, we analyzed 10 initial Rich Tobacco JUUL pods for each mouth piece. Total particulate matter (TPM) and liquid consumption were determined for a total of 20 fractions of 20 puffs each. Furthermore, to increase validity of our procedure, we analyzed 10 additional pods with the bought mouth piece in a different laboratory (lab B) for an inter-laboratory comparison.


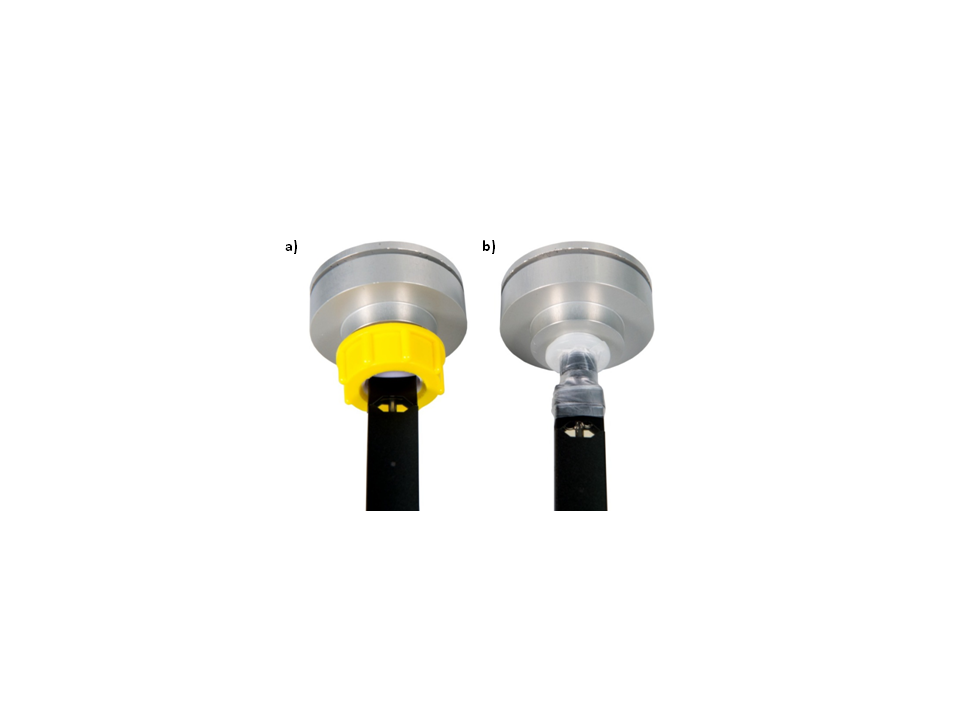


Figure 5. Two different mouth pieces for connection of the rectangular E-cigarette and the filter holder. Variant a) is commercially available, whereas variant b) is self-made with a heat-shrinkable tubing

The generated data for these two settings are displayed in Figure 6. For each collected fraction, the mean and standard deviation of the 10 analyzed pods were calculated. Four outcomes can be extracted: Firstly, the amount of consumed liquid correlates with the corresponding TPM collected. Secondly, the results from both laboratories are in good agreement. For the first 8 fractions (first 160 puffs), mean and standard deviation of TPM were 34 ± 6 mg and 32 ± 8 mg for lab A and lab B, respectively. Thirdly, in comparison of both mouth pieces in lab A, the commercially available mouth piece has led to more consistent results than the self-made variant. The self-made variant resulted in slightly higher but still acceptable results for the first 11 fractions. We assume that the connection between e-cigarette and mouth piece was slightly tighter for the self-made variant. As a consequence, we hypothesize that the activator within the e-cigarette, which is only activated by a sufficient flow, might have started the power supply of the coil a little bit earlier for each puff. The resulting longer heating duration together with the slightly higher flow may be the reason for the small increase in vapor generation. However, for future analyses of e-cigarettes with unusual and challenging shapes, self-fabrication of mouth pieces using heat-shrinkable tubing is possible for the determination of TPM and nicotine. Fourthly, the standard deviations are high and increasing towards the end of the analysis. This can only be explained with a different performance of the pods with their included coils and wicks.


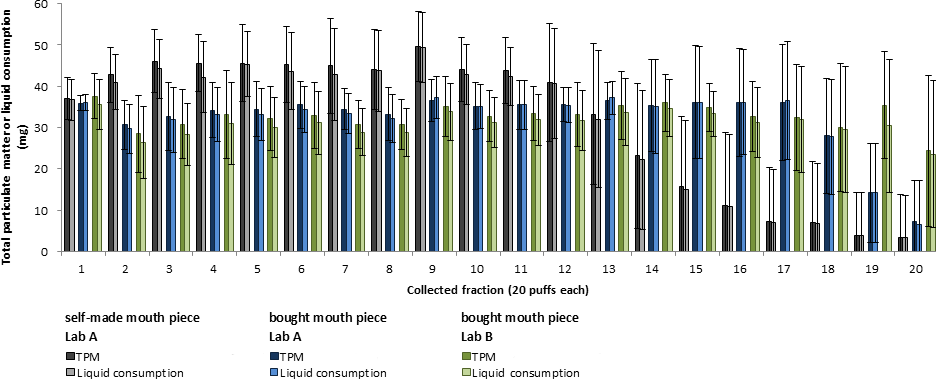


Figure 6. Mean and standard deviations of total particulate matter (TPM) and liquid consumption from 10 initial pods, analyzed in two different laboratories with two different mouth pieces

**Aerosol generation by modified JUUL version**

In lab A, TPM and liquid consumption of 6 modified Rich Tobacco JUUL pods with 9 and 18 mg/mL nicotine were analyzed using the commercially available mouth piece. TPM and liquid consumption are shown in Figure 7. With the modified pods, the deviation between fractions and the standard deviation between different pods decreased. This points to an improved consistency of vapor generation in the modified product.


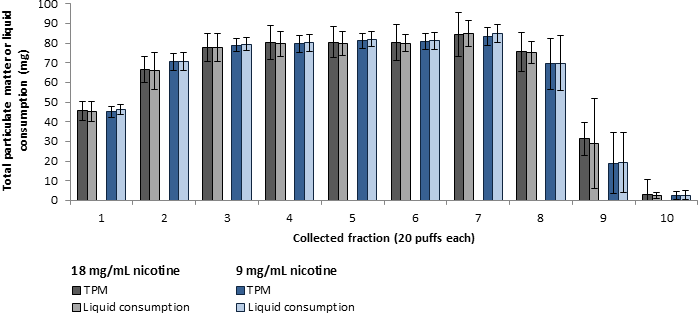


Figure 7. Mean and standard deviation of total particulate matter (TPM) and liquid consumption from 6 modified JUUL pods

**Comparison of JUUL versions and mouth pieces regarding cumulative aerosol generation**

Regarding the total liquid consumption over time in Figure 8, both JUUL variants and mouth pieces had a steady increase until about 600 mg were consumed. Afterwards, liquid was consumed much slower. The modified JUUL pods were nearly empty after 160 puffs, as already displayed in Figure 7. Pods of the initial version were nearly empty after roughly 300 or 340 puffs with the self-made and the bought mouth piece, respectively. As already visible in Figure 6, both mouth piece types show some differences in aerosol generation. However, the differences are small enough that the use of a self-made mouth piece is justified when a commercial option is not available. The modified JUUL version provides only half the number of puffs compared to the initial one. This could result in increasing in costs for the consumer.


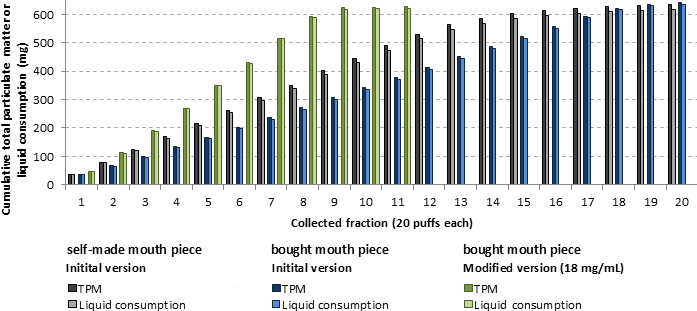


Figure 8. Cumulative total particulate matter (TPM) and liquid consumption from the mean of 6 modified JUUL pods and 10 initial JUUL pods, analyzed with different mouth pieces in lab A

**Correlation between measured and calculated nicotine levels in the aerosol**

The nicotine concentration in the aerosol was analyzed with GC/FID as described in the Methods section of the main manuscript. Additionally, the nicotine content can be calculated by multiplying the collected TPM with the nicotine concentration in the liquid (in mg/mg), as previously demonstrated by Talih et al. (Talih et al. 2017). To ensure quality, measured and calculated nicotine concentrations were compared using the data set from lab A for the initial Rich Tobacco pods with the commercially available mouth piece as an example. Only values above 10 mg TPM were included. As displayed in Figure 8, both ways to determine nicotine levels in the aerosol were in good agreement. Since a reliable quantification method was available in both laboratories, nicotine was measured with GC/FID. Nevertheless, the data imply that nicotine contents in the aerosol could be approximated only based on weighing of the e-cigarette, since the consumed liquid and the collected TPM have nearly the same mass as demonstrated in Supplementary Figures 6 and 7.


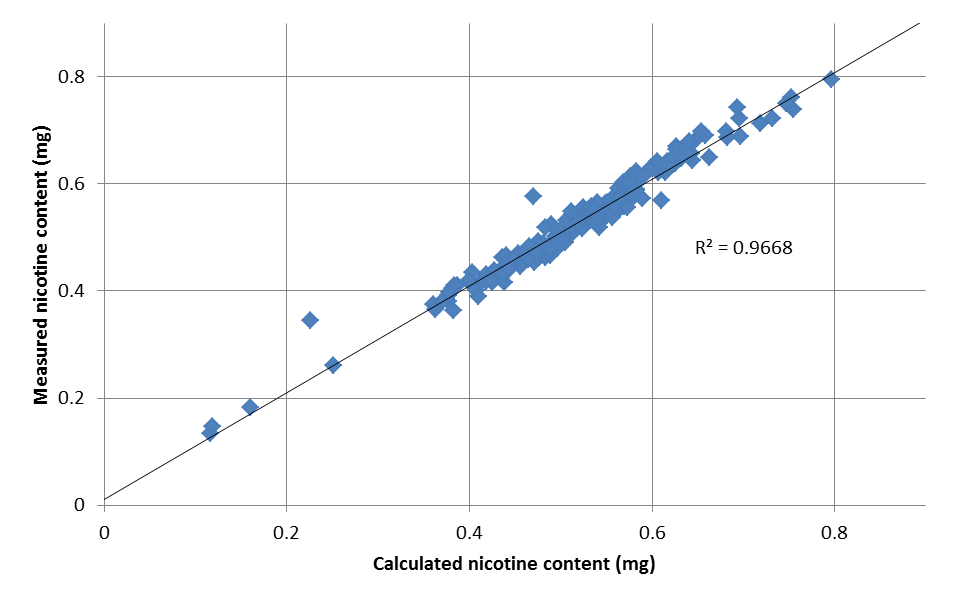


Figure 9. Correlation between measured and calculated nicotine contents in the aerosol

**Analytical limits of carbonyl compound quantification**

Limits of detection (LOD) and limits of quantification (LOQ) were estimated from the lowest standard (16.4 ng/puff) via the signal-to-noise ratio (S/N) and are presented in Table 2. S/N for LOD was 3 and for LOQ 5. Sample values between the lowest standard and LOQ were extrapolated; values between LOQ and LOD were set as the middle between LOQ and LOD.

**Table 2.** Limits of detection (LOD) and quantification (LOQ) for the analysis of carbonyl compounds calculated based on ng/puff

| Analyte | Limit of detection  (ng/puff) | Limit of quantification  (ng/puff) |
| --- | --- | --- |
| Formaldehyde | 0.5 | 0.9 |
| Acetaldehyde | 1.9 | 3.2 |
| Acetone | 1.3 | 2.2 |
| Acrolein | 1.1 | 1.9 |

**Continuity of carbonyl compound emission**

In terms of carbonyl compound emissions, a “dry puff” effect has been shown in e-cigarettes by some groups (Farsalinos and Gillman 2017; Hutzler et al. 2014). This effect describes that at the end of consumption, when the liquid is too low to sufficiently supply wick and coil, temperature and consequently formation of carbonyl compounds can increase, resulting in harsher emissions (Farsalinos and Gillman 2017). To find out whether this occurs with JUUL in the machine smoking set up as well, the ratio between the amount of carbonyl compounds and the liquid consumption was evaluated. An increasing ratio in combination with a decreasing amount of consumed liquid indicates a spike in carbonyl formation. These ratios per collected fraction and the according liquid consumption values are displayed for one exemplary pod (modified JUUL version) in Supplementary Figure 10. The limit of quantification per 40 puffs of each analyte was divided by the corresponding liquid consumption and included in the figure. For all experiments, carbonyl formation was low during the first 160 puffs. Enhanced carbonyl formation was detected after a strong decrease of the amount of evaporated liquid per puff was recorded (below 20 mg). This was due to exhaustion of the liquid reservoir. Although "dry puff" conditions can theoretically occur, hardly any aerosol is generated at that stage. The small number of repeats and especially the quantification close to the analytical limits should be noted. Values for carbonyl emissions in ng/puff in Table 1 in the main text include only the first 160 puffs to represent normal test conditions without spikes due to “dry puffing”.


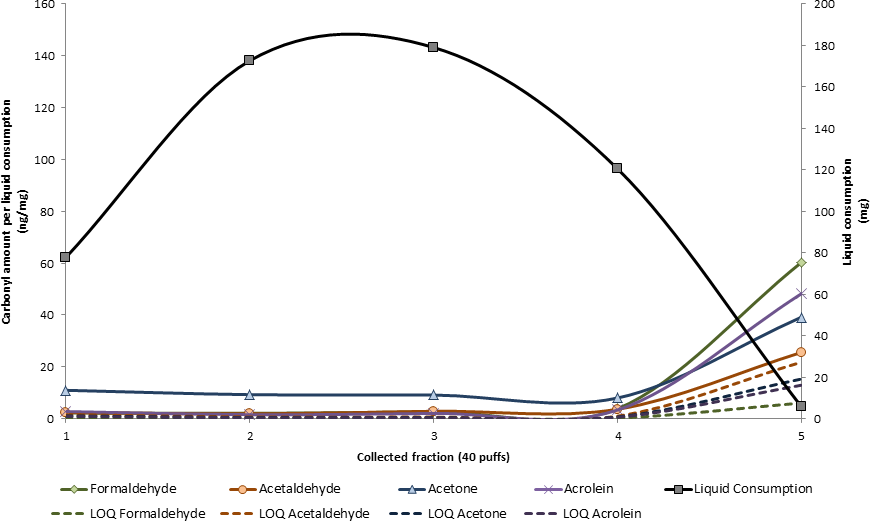


**Figure 10.** Carbonyl compound emissions by one modified JUUL pod in ng per mg consumed liquid (primary y-axis) and liquid consumption in mg (secondary y-axis) per collected fraction of 40 puffs each

**FT-IR spectra of wick material**

The differences in the used wick material are visible in the FT-IR spectra as seen in Figure 9. The American and the initial European JUUL versions deliver the same spectra, whereas the modified JUUL wick clearly consists of a different material. The spectrum of the modified JUUL wick in Figure 9a (Supplement) shows characteristic bands that are not present in the other two spectra: A wide band from 3600 to 3200 cm^-1^, originating from hydroxyl groups, stretching vibrations from aliphatic (C-H)-bonds (3000 to 2800 cm^-1^), and signals in the finger print region from 1500 to 1200 cm^-1^ (Hesse et al. 1991).


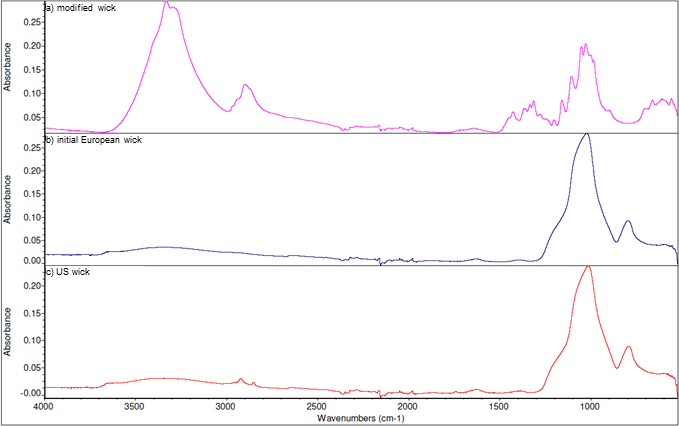


**Figure 11.** Attenuated total reflectance-Fourier-transform infrared (FT-IR) spectra of different wicks: a) modified JUUL, b) American-JUUL, and c) initial European JUUL

**References**

Dong C, Mei Y, Chen L (2006) Simultaneous determination of sorbic and benzoic acids in food dressing by headspace solid-phase microextraction and gas chromatography. J Chromatogr A 1117:109-114 doi:10.1016/j.chroma.2006.04.006

Dong C, Wang W (2006) Headspace solid-phase microextraction applied to the simultaneous determination of sorbic and benzoic acids in beverages. Analytica Chimica Acta 562:23-29 doi:10.1016/j.aca.2006.01.045

Farsalinos KE, Gillman G (2017) Carbonyl Emissions in E-cigarette Aerosol: A Systematic Review and Methodological Considerations. Front Physiol 8:1119 doi:10.3389/fphys.2017.01119

Hesse M, Meier H, Zeeh B (1991) Spektroskopische Methoden in der organischen Chemie. Georg Thieme Verlag, New York

Hutzler C, Paschke M, Kruschinski S, Henkler F, Hahn J, Luch A (2014) Chemical hazards present in liquids and vapors of electronic cigarettes. Arch Toxicol 88:1295-1308 doi:10.1007/s00204-014-1294-7

Talih S, Balhas Z, Salman R et al (2017) Transport phenomena governing nicotine emissions from electronic cigarettes: model formulation and experimental investigation. Aerosol Sci Technol 51:1-11 doi:10.1080/02786826.2016.1257853
